# A Review of Environmental-Based Community Interventions

**Published:** 2011

**Authors:** Traci L. Toomey, Kathleen M. Lenk

**Keywords:** Alcohol and other drug use, problematic alcohol use, risk and protective factors, prevention, preventive intervention, individual-based prevention, environmental-based community intervention, alcohol policy, Communities Mobilizing for Change on Alcohol, Community Prevention Trial, Sacramento Neighborhood Alcohol Prevention Project, Saving Lives, Operation Safe Crossing, Fighting Back

## Abstract

Alcohol use and related problems can be influenced by a wide variety of prevention interventions, including efforts that focus on changing the community alcohol environment—for example, by reducing underage access to alcohol, decreasing alcohol availability among adults, and increasing awareness of alcohol-related issues. Examples of environmental-based community interventions that focus on reducing alcohol use and related problems are Communities Mobilizing for Change on Alcohol, the Community Prevention Trial, the Sacramento Neighborhood Alcohol Prevention Project, Saving Lives, Operation Safe Crossing, and Fighting Back. Evaluations of these programs found that programs that change the community environment can reduce alcohol use and related problems among both youth and adults, even in communities with relatively low readiness to address alcohol issues. Research also has identified particular settings and situations where alcohol environmental changes are particularly needed as well as factors influencing the effectiveness of certain strategies. Despite the progress made, additional questions still need to be addressed in future research to maximize the benefits associated with environmental-based community interventions.

Alcohol use and related problems are affected by a myriad of both individual- and environmental-level risk factors (Wagenaar and Perry 1994). Prevention interventions that focus solely on individual-level risk factors generally do not affect community-level outcomes and need to be reinforced by changes in the broader environment in order to achieve sustained population-level effects (Room et al. 2005; Wagenaar and Perry 1994). In contrast, research suggests that prevention interventions that focus solely on altering the alcohol-related environment can be effective in reducing alcohol-related problems at a population level on their own (Babor et al. 2003).

Much of the research evidence demonstrating the effectiveness of changing the alcohol-related environment comes from studies of State-level alcohol policies (e.g., the age 21 minimum legal drinking age and alcohol excise taxes) (Elder et al. 2010; Wagenaar and Toomey 2002; Wagenaar et al. 2009). However, interventions that change the alcohol environment at the community level also can be effective (Hingson and Howland 2002). This review summarizes interventions that focus primarily on changing the community alcohol environment.

## Alcohol-Related Community Intervention Studies

As described by Holder (2002, p. 906), “a ‘community’ is viewed as a set of persons engaged in shared social, cultural, political, and economic processes.” An environment-based community intervention focuses on modifying this system such that the likelihood of alcohol use and/or related problems is reduced. Some of the issues addressed in these interventions include the following:

- Reducing underage access to alcohol from commercial (e.g., bars, restaurants, and liquor stores) or social providers (e.g., friends, parents, and coworkers);
- Decreasing alcohol availability among adults (e.g., by promoting responsible service of alcohol and increasing enforcement of alcohol-control policies);
- Increasing enforcement of drinking-and-driving laws; and
- Implementing awareness campaigns or expanding media coverage to increase awareness of and focus on alcohol-related issues.

No curricula or manuals exist that specify how to make these changes in the community. Moreover, each community is unique, complex, and not always predictable. But it is clear that without changing the community system—or the environmental determinants of behavior—the community system will continue to generate more individuals who need to be educated or treated in order to reduce alcohol use and related problems (Holder 2002; Wagenaar and Perry 1994).

Six environment-based community interventions that specifically focus on reducing alcohol use and alcohol-related problems have been developed and evaluated and are reviewed here, including Communities Mobilizing for Change on Alcohol (CMCA), the Community Prevention Trial (CPT), the Sacramento Neighborhood Alcohol Prevention Project (SNAPP), Saving Lives, Operation Safe Crossing, and Fighting Back.

CMCA focused on underage youth (i.e., those under the legal drinking age of 21). From 1993 to 1994, the program used a grassroots community-organizing approach to implement multiple strategies to prevent underage individuals from obtaining alcohol in order to ultimately reduce alcohol use and related problems (Wagenaar et al. 2000*a*). Communities located in Minnesota and Wisconsin were selected for the study on the basis of their size and geographic location—not on the basis of their readiness to work on this issue. Seven of these communities were randomly assigned to the intervention condition and eight to the comparison condition. In the intervention communities, various institutional policy and practice changes were implemented to reduce access to alcohol, such as alcohol compliance checks by law enforcement agencies at bars and liquor stores and enforcement of stricter drinking policies at community festivals (for a full description of the CMCA intervention, see Wagenaar et al. 1999). Each community identified its own unique set of strategies, guided by a list of promising strategies targeting youth access to alcohol. The evaluation of the project found that 18- to 20-year-olds in intervention communities were less likely than their peers in the comparison communities to try to buy alcohol, drink in a bar, consume alcohol, or be arrested for driving under the influence. Drinking behavior among 12th graders, however, was not affected (Wagenaar et al. 2000*a*, *b*).

The CPT was conducted in three matched intervention communities in California and South Carolina from 1992 to 1996 (Holder et al. 2000). The communities were selected on the basis of whether coalitions already existed that were interested in the proposed comprehensive strategies. The intervention targeted underage drinkers as well as the general population and included five evidence-based strategies that each community was asked to implement—community mobilization, responsible beverage-service training at bars, limitation of access to alcohol through zoning, compliance checks to prevent sales to underage youth, and sobriety checkpoints to prevent drinking and driving. A time-series evaluation found declines in alcohol sales to minors, self-reported drinking and driving, nighttime traffic crashes resulting in injuries, traffic crashes in which the driver had been drinking, and assault injuries observed in emergency departments in intervention communities relative to comparison communities (Holder et al. 2000).

SNAPP, which was implemented between 2000 and 2003, aimed to reduce alcohol access, drinking, and related problems among underage youth and young adults (i.e., people ages 15 to 29) (Treno et al. 2007). The project adapted the CPT model for application in two low-income, predominantly ethnic-minority neighborhoods, with the Sacramento community at large serving as a comparison. Intervention communities were selected on the basis of their demographics as well as the existence of community-based organizations that were “sympathetic—but inexperienced in—environmental prevention” (Treno et al. 2007, p. 198). Communities were expected to implement five intervention components, including community mobilization, community awareness, responsible beverage-service training, underage-access law enforcement, and intoxicated-patron law enforcement. Evaluations of the program found significant reductions in assaults as reported by police, calls to emergency medical services for assaults, and motor vehicle crashes. However, no changes were found in propensity of sales to underage or intoxicated patrons, emergency calls for alcohol and other drug problems or suicides, or police reports for public drunkenness (Treno et al. 2007).

Saving Lives, which specifically targeted traffic-related outcomes, was implemented in six Massachusetts communities (chosen among a group that applied for funding through the project) beginning in March 1988. Intervention staff organized city departments and private citizens in the communities to reduce alcohol-impaired driving, related driving risks, and traffic deaths and injuries (Hingson et al. 1996). Intervention strategies varied by city and included efforts such as police training, beer-keg registration, public education efforts, and media and awareness campaigns. Evaluation results show that relative to the rest of Massachusetts during the 5 program years, total and alcohol-related fatal crashes declined significantly in the program cities (Hingson et al. 1996).

Operation Safe Crossing, which also is known as the Border Binge Drinking Reduction Program, initially was developed by a local consortium in San Diego, California, in 1997 to reduce the number of impaired drivers crossing the border from Mexico to the United States on weekend evenings. The principal initial effort focused on strengthening enforcement of drinking-and-driving regulations through measures such as special patrols, sobriety checkpoints, foot patrols monitoring the pedestrian-crossing area, and media campaigns (Voas et al. 2002). Evaluations of the program showed decreases in the overall number of young people returning to the United States and in the ratio of had-been-drinking crashes to had-not-been-drinking crashes among drivers aged 16 to 20 years (Voas et al. 2002).

The Fighting Back project included 14 communities (across 11 States and Washington, DC) that, from 1992 to 1997, received funds to reduce substance abuse and related problems. An evaluation was conducted of five of these communities that implemented at least eight interventions to reduce alcohol availability or increase substance abuse treatment services (Hingson et al. 2005). Commonly implemented interventions among these five communities included increased access to treatment, emergency-department–based screening and referral, alcohol compliance checks, responsible beverage-service training, revised ordinances on public consumption or beverage sales, and actions to address problematic outlets or outlet density. A quasiexperimental design matched each of the five communities to two or three other communities of similar demographic composition in the same State. Relative to the comparison communities, the intervention communities experienced significant declines in alcohol-related fatal crashes (Hingson et al. 2005).

Although only a relatively small number of studies have evaluated environmental-based community interventions, they provide evidence that this approach is promising for effectively reducing alcohol-related problems. All of the studies showed effects on drinking-and-driving–related outcomes. Several similarities between the interventions existed with respect to the components implemented. For example, all interventions included at least some strategies to reduce the availability of alcohol for underage youth and/or the adult population. Furthermore, all interventions involved mobilization of community citizens, leaders, and institutions; focused primarily on changing the community environment; and implemented multiple changes rather than a single change to the alcohol-related community environment.

However, several differences also existed among these interventions. First, whereas CMCA only focused on underage youth, the other interventions included strategies targeting the general adult population as well as youth. Second, the selection criteria for communities participating in each intervention differed, which had the potential to affect the likelihood of the environmental changes being made. For example, CMCA communities were selected solely on the basis of research evaluation needs (i.e., size and geographic location), whereas CPT communities were selected on the basis of their explicit readiness to implement environmental alcohol interventions. Third, the interventions varied in how the implemented strategies were selected. Although communities participating in CPT and SNAPP were asked to implement preselected, evidence-based strategies, communities participating in other interventions could choose from a range of strategies, with each community implementing a unique combination of environmental strategies. Finally, the interventions differed in whether they used environmental strategies targeting alcohol use, specific alcohol-related problems (e.g., drinking and driving), or a combination of these two.

In addition to these community-level environmental strategies, environmental strategies at the State level, on college campuses, and in schools also can be effective in reducing alcohol use and related problems (e.g., Hawkins et al. 2009; Perry et al. 2002; Saltz et al. 2009, 2010; Wagenaar et al. 2006; Weitzman et al. 2004). The findings of this additional research are summarized in the articles by Fagan and colleagues (pp. 167–174) and Saltz (pp. 204–209) in this issue.

## Implications for Practitioners

The studies reviewed in the previous section provide evidence that changing the community environment can reduce alcohol use and related problems among youth and adults. They also suggest that environmental changes can be achieved in communities with varying degrees of readiness to address alcohol issues by implementing environmental change. It seems, however, that multiple community-level changes may be needed to reduce alcohol use and related problems; in contrast, studies of State-level alcohol policies have found that change in a single alcohol policy, such as the age 21 minimum legal drinking age or an increase in alcohol excise taxes, can be sufficient. Researchers have not identified specific combinations of environmental strategies necessary for preventing alcohol-related problems. However, the studies suggest that it is important to consider which environmental strategies are most appropriate for the targeted outcome and population. For example, CMCA was effective in reducing alcohol use among 18- to 20-year-olds but not among 12th graders; a possible explanation for these findings is that the strategies implemented by CMCA communities may have targeted sources of alcohol more commonly used by the older age-group (e.g., licensed alcohol establishments) (Wagenaar et al. 1999, 2000*a*, *b*). Taken together, however, the studies suggest that it is possible to change the alcohol environment both in communities that are selected on the basis of their readiness for change and in those that may not be ready for change.

The research literature clearly supports the conclusion that changing the community environment can reduce alcohol use and related problems, and although communities may have limited guidance about specific environmental strategies, they can draw on some studies to guide their efforts. For example, researchers have identified particular settings and situations where alcohol environmental changes are needed, such as at alcohol establishments, community festivals, and professional sport stadiums where studies have found that the likelihood of illegal sales to obviously intoxicated patrons is high (Lenk et al. 2006; Toomey et al. 2005, 2008). Other investigators have determined how specific strategies can be most effective; for example, alcohol compliance checks were found to be effective if implemented at least every 3 months (Wagenaar et al. 2005). To implement the most effective strategies possible, ongoing and future environmental-based community interventions therefore should draw from the most recent research studies.

## Implications for Future Research

Several additional questions remain that can guide future research on environmental-based community interventions to reduce alcohol use and related problems:

- Which environmental interventions, implemented alone or in combination, are most effective? None of the studies conducted to date were able to determine which specific individual strategies or combinations of strategies mediated the observed outcomes. Therefore, studies are needed to assess which individual or combinations of environmental-change strategies are most effective in reducing alcohol use and related problems.
- Are effects of community interventions strengthened when combined with individual-level interventions? Although evidence indicates that environmental changes alone are sufficient to produce reductions in alcohol use and related problems, researchers have not explicitly assessed whether adding individual-level interventions would enhance the effects of environmental-based community interventions.
- What is the optimal way to implement specific environmental interventions? Community groups commonly want to know how to implement specific types of environmental changes, and more research is needed to guide implementation of the wide range of environmental strategies available.
- How should research findings be disseminated and what type of technical assistance is needed? Researchers need to identify the most effective mechanisms to disseminate their findings to guide environmental-based community interventions. Moreover, the type of assistance that is required to ensure these environmental changes are implemented needs to be determined.
- What would be the results if these studies were replicated today? Most of the environmental-based community interventions described in this article were conducted during the 1980s and 1990s, when few communities were focusing on alcohol-related problems and limited information was available about many of the specific environmental strategies implemented. More current evaluations are needed of environmental-based community interventions that are guided by the most current research findings on specific alcohol environmental strategies.
